# *In silico* Mapping of Protein Unfolding Mutations for Inherited Disease

**DOI:** 10.1038/srep37298

**Published:** 2016-12-01

**Authors:** Caitlyn L. McCafferty, Yuri V. Sergeev

**Affiliations:** 1 Ophthalmic Genetics and Visual Function Branch, National Eye Institute, NIH, Bethesda Maryland, 20892, USA

## Abstract

The effect of disease-causing missense mutations on protein folding is difficult to evaluate. To understand this relationship, we developed the unfolding mutation screen (UMS) for *in silico* evaluation of the severity of genetic perturbations at the atomic level of protein structure. The program takes into account the protein-unfolding curve and generates propensities using calculated free energy changes for every possible missense mutation at once. These results are presented in a series of unfolding heat maps and a colored protein 3D structure to show the residues critical to the protein folding and are available for quick reference. UMS was tested with 16 crystal structures to evaluate the unfolding for 1391 mutations from the ProTherm database. Our results showed that the computational accuracy of the unfolding calculations was similar to the accuracy of previously published free energy changes but provided a better scale. Our residue identity control helps to improve protein homology models. The unfolding predictions for proteins involved in age-related macular degeneration, retinitis pigmentosa, and Leber’s congenital amaurosis matched well with data from previous studies. These results suggest that UMS could be a useful tool in the analysis of genotype-to-phenotype associations and next-generation sequencing data for inherited diseases.

A mutation in a normal DNA sequence has the potential to cause a genetic disorder. Such inherited disorders often involve a combination of genetic and environmental factors. Thousands of human diseases are caused by single-gene defects, many of which involve the eye. In some cases, genetic mutations cause changes at the protein level, affecting protein structure, stability, and function. Protein polypeptides fold into their native conformations, which are maintained by weak, noncovalent forces, and the polypeptides undergo various posttranslational modifications and the formation of disulfide bonds. In the endoplasmic reticulum (ER), protein disulfide isomerase (PDI), a cellular chaperone with a foldase function, catalyzes the formation of disulfide bonds to maintain the native protein fold^1^,^2^. PDI then catalyzes the oxidation and reshuffling (isomerization) of disulfides in the targeted motif of the substrate protein^3^. Because the “energy surface” or “landscape” is encoded by the amino-acid sequence^4^, the protein folds through several competing pathways into intermediate non-native structures. These structures progress through decreasing free energies until they achieve a conformation with the lowest energy, forming a globular protein with native interactions^5^. Disease-causing mutations might inhibit the pathway to the lowest energy conformation and cause the protein to remain in a non-native conformation with a higher free energy. The unfavorable free energy of a non-native protein lowers the probability of partly folded states and increases the cooperativity of the unfolding transition^6^. Non-native proteins never achieve the lowest energy conformation. Misfolded, non-native proteins can be processed by chaperone-mediated autophagy and/or degraded in the cytosol through the ubiquitin–proteasome pathway^7^. These possibilities are supported by observations of proteins modified by genetic mutations and expressed in cell cultures; the modified protein bands are absent from SDS–PAGE or native gels and are similar to the bands of ‘null’ protein mutations^8^,^9^. The formation of non-native proteins is controlled by the unfolded protein response (UPR), a response to the accumulation of unfolded/misfolded protein in the ER, which increases correct protein folding and improves the ER-associated degradation of misfolded proteins^9^,^10^.

The effects of missense mutations and their relation to disease are still not well understood. It has been suggested that mutations that cause changes in biophysical characteristics, such as charge, hydrophobicity, and geometry, tend to lead to disease^11^,^12^. Disease-causing missense mutations tend to disturb hydrogen bonding networks and disulfide and salt bridges, thus altering the native state of the protein.

Khan and Vihinen have previously evaluated the performance of computational protein stability predictors^13^. Here, the performance of 11 online stability predictors was analyzed: CUPSAT, Dmutant, FoldX, I-Mutant2.0, I-Mutant3.0, MultiMutate, MUpro, SCide, Scpred, and SRide. In total, 1,784 missense mutations in 80 different proteins were analyzed. The mutations were categorized as stabilizing, neutral, or destabilizing. The programs all predict stability differently, so Khan and Vihinen established universal parameters to compare the predictors, including accuracy, sensitivity, specificity, and the Matthews correlation coefficient (MCC). For the parameters used above, they concluded that the most reliable predictors for structures were I-Mutant3.0, Dmutant, and FoldX. For this reason, we chose to use FoldX ΔΔG in our calculations of the unfolding propensities of each mutation.

One important application is understanding the role of disease-causing mutations in the formation of clinical phenotypes. Computational predictive methods may be useful in precision medicine. The use of computational tools in the precision medicine initiative is pivotal in dealing with large datasets^14^,^15^,^16^,^17^. The primary areas of focus in strides towards precision medicine include processing large-scale robust genomic data, interpreting the functional effects and effects of genetic variations, integrating systems data to relate complex genetic interactions to phenotype, and translating discoveries into medical practice^14^.

Several tools, including the SNPeffect database, are able to predict the functional consequences of missense mutations in protein structures^18^,^19^,^20^,^21^,^22^,^23^. Many of these methods depend on evolutionary sequence conservation. These methods were tested in a study where the predicted results were compared to the effects of missense mutations^24^. The mutations were measured in homozygous mice *in vivo*, and the mice were monitored for a loss-of-function phenotype. The study found that because many of these methods depend on evolutionary conservation, they were unable to accurately predict the effects of *de novo* mutations. This inability is a problem in differentiating clinically relevant mutations from neutral mutations. A method that can predict the functional consequences of *de novo* mutations could greatly assist in handling novel mutations.

Another approach is to link genetic changes at the level of the protein atomic structure with the disease phenotype. The severity of the mutational effect could be evaluated based on changes in the energetics of the protein atomic structure and the physical properties of amino acid residues^25^. This function could be used to demonstrate genotype-to-phenotype relationships in monogenic disease^26^,^27^,^28^. However, evaluating mutation effects using free energy changes is difficult for several reasons. A direct molecular dynamics calculation of the Gibbs free energy changes is a very slow process and difficult to implement for large volumes of patient mutation data. An effective alternative to this process is the use of semi-empirical methods, such as FoldX^29^. However, energy calculations are inadequate for the quantitative prediction of genotype-to-phenotype relationships. For example, there is no clear threshold that indicates how large the folding energy changes should be for a mutant with a complete loss of protein activity. In addition, our experience indicates that it is difficult to use free energy to evaluate the genome severity for a recessive genetic disease in which several alleles are affected by genetic changes. Therefore, measuring the protein unfolding that results from missense mutations, instead of measuring free energy changes, has the ability to be a better predictor for diseases associated with protein instability.

Changes in protein folding could be characterized by the fraction of unfolded molecules or the unfolding propensity, which is determined using the linear extrapolation model from the experimentally obtained normalized sigmoidal unfolding curve^30^. In our work, unfolding propensity is evaluated *in silico* based on the atomic protein structure and the molecular modeling of the impact of the missense mutation on this structure. The unfolding fraction was determined from the free energy changes associated with the protein transition from the folded to unfolded thermodynamic state. Using the unfolding propensity, we have created the unfolding mutation screen (UMS). UMS calculates the unfolding propensity, a measure of the ability of a protein to fold properly, for all possible missense mutations that a structure can undergo based on the protein atomic structure. The unfolding values are derived from the free energy changes between the mutant and wild-type protein structures. These data are then transformed into a series of easily read maps that can be selected based on the user’s purpose.

Our unfolding calculations were verified by experiments on 1391 mutant variants from 16 protein crystal structures. The analysis showed that the proteins from the validation dataset had an average of 77.9 ± 9.1% correct matches between the experimental and computed unfolding. The method was then applied to analyze the mutations and critical residues in genetic eye disorders, such as age-related macular degeneration (AMD), autosomal dominant Retinitis Pigmentosa (adRP), and Leber’s congenital amaurosis (LCA).

## Results

### Development of the *in silico* unfolding program

**Figure 1:**
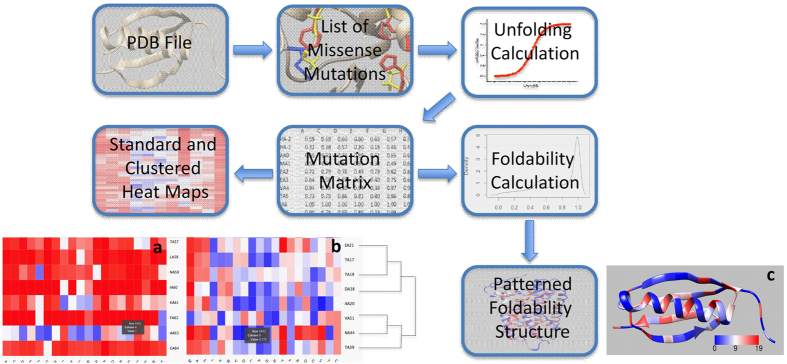
Schematic illustrating the workflow of the UMS process. The input for the program is a PDB file. A list of all possible missense mutations is generated for the specific protein. Next, the unfolding propensity is calculated from the FoldX free energy change between the mutant and wild-type protein structure. The data are then sorted into a mutation matrix, which is used to construct the standard unfolding heat map (**a**). Adjacent to that map, the clustered unfolding heat map was built using a dendrogram to track the grouping of the data (**b**). The mutation matrix is also used in the foldability calculation described in the Methods section. Finally, these foldability values are used to color the foldability structure (**c**).

The UMS code was developed using the Python, Bash, and R languages. As shown in the flowchart (Fig. 1), each residue of the atomic protein structure was mutated to 19 different amino acids and one identity mutation. For each mutation, the free energy changes were calculated *in silico* using FoldX^29^ and converted to the mutation unfolding propensities. The properties of the mutations at each position in the structure were characterized using standard and clustered unfolding heat maps. The standard unfolding heat map shows the unfolding data for each residue from the amino acid sequence of the protein. The clustered heat map uses an agglomerative hierarchical clustering method to group residues with similar mutational effects.

In addition, we developed a Python code for the Chimera viewer to show critical residues in protein folding. For this purpose, we use the foldability parameter, which is a sum of severity-weighted unfolding propensities for the 20 mutations generated at the same residue position of the protein structure. Foldability ranges from 0–19, where 0 represents mutations that favor a stable protein structure, and 19 represents mutations that favor unfolding in this position. Residues with the highest foldability were considered critical for protein folding.

The *in silico* unfolding propensities were validated by the unfolding propensities obtained from experimental free energy changes for 1,391 mutations from 16 proteins. Supplementary Figure S1 shows the corresponding match matrices for each protein; the values in grey along the diagonal display the largest numbers, indicating a preference for matches over mismatches.

Table 1 summarizes these results by assigning 2 different scores to the proteins. The percent matching quantifies the data from the match matrices. The protein with the lowest percent matching, 66.7%, was alpha spectrin. The best percent matching score belonged to ribosomal protein S6, with a value of 100%. Therefore, the proteins exhibited an average match of 77.9 ± 9.1% between the experimental and computational unfolding propensities. The fit score (formula 1) also showed the agreement between the experimental and computational unfolding propensities. The worst fit was tryptophan synthase, with a value of 0.347, while the best fit score belonged to azurin, with a value of 0.085. This result shows that the *in silico* unfolding propensities agree with the fractions of unfolded molecules obtained from equilibrium unfolding experiments. Supplementary Table S2 lists the critical residues for each of these structures. Based on the heat maps, we see that many of the critical residues in these structures are glycine, proline, and cysteine residues.

To analyze disease-causing mutations in rhodopsin, complement factor H (CFH), and retinal pigment epithelium protein 65 (RPE65), the homology models of these proteins were generated as described in the Methods section. The protein structures were subjected to internal control for identical changes, where each residue in the sequence was mutated to itself, and the unfolding propensity was calculated (Supporting Table S1). From our observations, the control had higher values when the protein structure model had poor stereochemistry. The homology models of rhodopsin, RPE65 and CFH had means of 0.49 ± 0.02, 0.49 ± 0.04, and 0.56 ± 0.18, respectively. For each of the proteins, the data were statistically significant (p-value < 2.2 × 10^−16^), indicating that the homology models showed good stereochemistry. Afterward, ~7000 rhodopsin, ~24,000 CFH and ~21,000 RPE65 mutations were obtained using UMS to identify the critical residues for proper protein folding (52 for rhodopsin, 235 for CFH, and 70 for RPE65). The effects of these residues were compared with known disease-causing missense mutations (83.3% of 90 for rhodopsin and 71.9% of 32 for CFH) or recombinant mutant variants (38 for RPE65) and indicated roles in protein unfolding. The corresponding UMS heat maps and foldability structures are available for RHO, CFH, and RPE65 along with 12 other proteins from McCafferty & Sergeev (ref. 31).

### Human rhodopsin: mutation classification

Here, human rhodopsin, a protein that has been studied extensively for its relationship to retinitis pigmentosa (RP), was analyzed^32^,^33^,^34^,^35^,^36^,^37^,^38^,^39^,^40^. Rhodopsin is a membrane protein found in retinal rods. The conformational change from 11-cis-retinal into all-trans-retinal in rhodopsin is an essential step of the visual cycle. Significant clinical data exist regarding the effects of missense mutations of rhodopsin^36^. Previously, rhodopsin mutations were divided into several classes by protein function, including class I and II mutations^41^. For class I mutations, the protein folds normally but is not transported to the outer segment, while for class II mutations, the protein is either retained in the ER, fails to reconstitute with 11-cis retinal, or accumulates in both the ER and the plasma membrane^41^,^42^. For rhodopsin, the phenotype data for class II mutations were compared with the *in silico* unfolding values. The heat maps showed that the areas of the least severe mutations occurred in the range of residues 240–250 and 340–348, which are exposed to the cytoplasm (Supplementary Figure S2).

**Figure 2:**
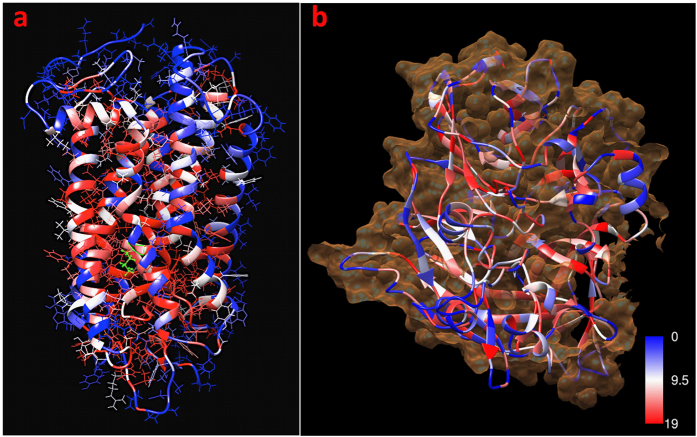
Foldability coloring for the rhodopsin and RPE65 protein structures. The red residues represent the wild-type residues that exhibit the most severe unfolding effects (high foldability) when mutated, while the blue residues maintain their stability (low foldability) when mutated. (**a**) Side chains of rhodopsin residues with the active site K296 highlighted in green to show the critical residues of the rhodopsin structure surrounding the active site. (**b**) The surface of RPE65 is shown in orange around the ribbon structure, revealing severe unfolding within the β-sheets. The foldability coloring was obtained in the program Chimera using the Python script described in the Methods section.

**Figure 3:**
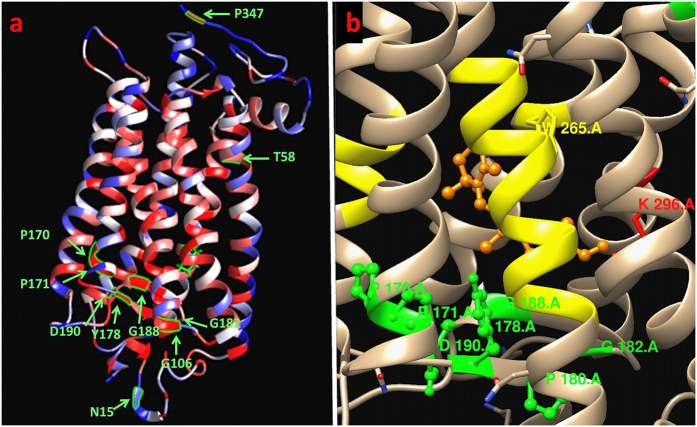
Foldability structure of rhodopsin displaying the mutations from Table 2. (**a**) The amino acid residues affected by the mutations are shown in green. All mutations except N15S have high foldability, indicating residues that are essential for proper protein folding. (**b**) The majority of mutations with high foldability values lie near the retinal chromophore binding site. The retinal binding residues and N6-(retinylidene) lysine (K296) are yellow and red, respectively. The retinal molecule is shown in orange.

Table 2 shows the relationship between our calculated unfolding values and the phenotype data^43^. The unfolding values were obtained from the unfolding heat map and used to predict the classes of these mutations. In general, a majority of the critical residues were in the intradiscal or transmembrane regions of rhodopsin, while the part of the molecule exposed to the cytoplasm showed fewer critical residues (Fig. 2a). The region of the structure is also displayed in Fig. 3, in which we see that 9 of the 10 mutations exist in either the intradiscal or transmembrane region. UMS indicated that 90% of the listed mutations exhibit severe destabilization. For the N15S mutation, we see that the age of onset for symptoms is later in life than for many of the other mutations, which is consistent with the unfolding propensity value of this mutation. P23 exhibited a foldability value of 18.98, indicating that mutations at that location had a strong unfolding effect. The frequently occurring P23H mutation has been shown to destabilize the rod photoreceptor disk membrane and could cause retinal degenerative disease^44^. The T58R mutation also shows a later average age of onset than the other mutations and has a large standard deviation, which accounts for the later age of onset. The P347L variant, the most frequent mutation in rhodopsin, accounts for 33.3% of all mutant alleles in the Spanish adRP cohort^43^, and P347L was predicted to be an unfolding mutation (class II). Supplementary Table S3 summarizes the genotype data for 90 rhodopsin mutations related to disease. For these 90 mutations, the larger unfolding propensities correspond to the class II mutations, as expected, with an average unfolding propensity of 0.88 ± 0.24.

### Critical residues in CFH sushi domains

**Figure 4:**
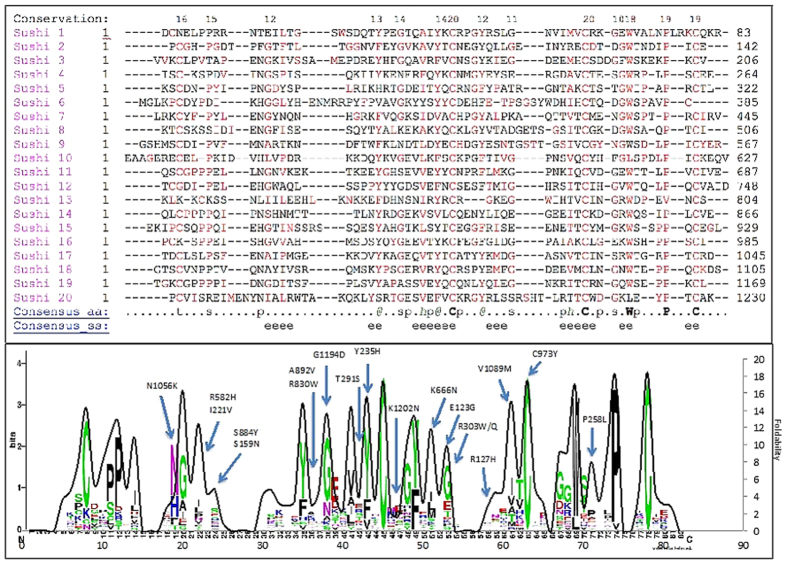
Multiple sequence alignment showing the conservation of the unfolding propensities of the complement factor H sushi domains. The 20 sushi domains were separated, and the sequences of each domain were compared by Promals3D multiple sequence alignment. (**a**) The alignment of the sushi domains shows significant conservation for the residues with high foldability values. These residues, which could cause protein unfolding when mutated, are colored red. (**b**) From the sequence alignment shown in (**a**), the logos of the conserved residues (http://weblogo.berkeley.edu/logo.cgi) were calculated and superimposed on the average unfolding values, shown by the black curve. The highest peaks of the curve correspond to the positions of the red residues from the sequence alignment in (**a**). The previously described genetic mutations^64^ are labeled on the plot.

**Figure 5:**
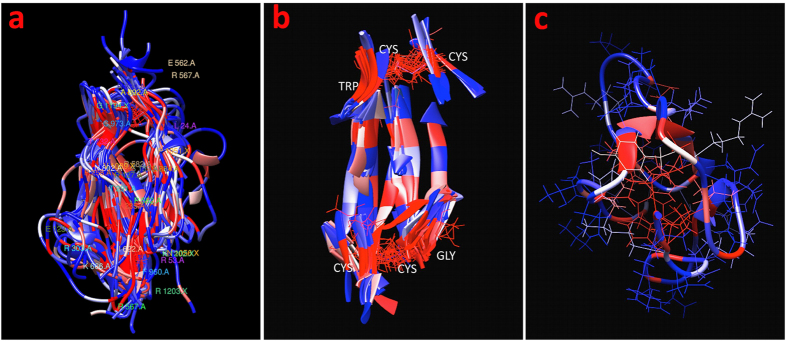
Foldability coloring of the complement factor H sushi domains. High-foldability residues are considered critical and are colored in red. Low-foldability residues are shown in blue. (**a**) The sushi domains are superimposed based on their structural alignment. (**b**) Structural alignment of β-strands in the 20 aligned sushi domains. (**c**) A side view of sushi domain 5 with the side chains of the residues. The critical residues are concentrated towards the hydrophobic core. The foldability coloring was obtained in the program Chimera using the Python script described in the Methods section.

We analyzed the role of unfolding in the sushi domains of the CFH protein by separating the CFH structure into its 20 sushi domains. The amino acid sequences of these domains were aligned by multiple sequence alignment (Fig. 4a). Based on this alignment, the logo plot for the conservation of the residues was obtained, and the average foldability of the 20 domains was plotted as a solid black line (Fig. 4b). The foldability curves, the expected locations of sensitive residues for mutation-caused unfolding, and the sequence conservation showed good agreement for all sushi domains. In addition, the atomic structures of the 20 sushi domains were superimposed upon each other (Fig. 5a), and the majority of the red-colored residues remained consistent along each of the domains. Figure 5c shows a view of the hydrophobic core, illustrating the large concentration of red residues occurring in this area and the blue color of the external residues. In Fig. 5b, the beta sheets in the domains are shown to highlight the striping effect of the red- and blue-colored residues along the sheets, which indicates a pattern of conservation at residue positions that are potentially sensitive for the mutation-caused protein unfolding. Finally, the AMD-related mutations in CFH were compared to the *in silico* unfolding propensities. For the 30 disease-related mutations, 70% showed a destabilizing unfolding effect, and 47% showed severe destabilization. The mutations that showed severe destabilization are labeled in Fig. 4b. Each sushi domain contains 4 cysteine residues and 2 disulfide bonds. All of the cysteine residues in the domains displayed foldability values of ~19.0. This result is consistent with the logo plot, which shows that the cysteine residues are highly conserved within the domains (Fig. 4b).

This result was confirmed by the mutation scan for TIMP3 and rhodopsin, where cysteines involved in the disulfide bonds exhibit severe unfolding with a foldability > 18 (Supplementary Figure S3). Glycine and proline residues are also essential for the structural characteristics of proteins due to their flexibility. In the TIMP3 structure, 71% of the glycine residues were critical residues. In rhodopsin, 57% of glycine and 60% of proline residues were critical residues. In CFH, 79% of glycine and 74% of proline residues were critical. CFH also showed that glycine and proline were highly conserved among the sushi domains.

### RPE65 catalytic activity

Protein catalytic activity is maximized for the properly folded protein and decreased for the misfolded protein. To understand the relationships between enzymatic activity and unfolding propensity, mutations affecting the normal function of RPE65 were analyzed. In the beta propeller structure of RPE65, the amino acid residues located in beta strands are critical to proper protein folding (Fig. 2b). The isomer hydrolysis activities for 11 RPE65 mutations in LCA patients^45^ were compared with our unfolding values using a binary system, where the mutations were either destabilizing or stabilizing. This analysis showed 73% agreement for the mutations based on this classification (Supplemental Table S4). Here, for the A434V mutation, we see an increase in activity to 110% of the wild-type activity. Our unfolding value agrees; the unfolding value of 0.01 indicates a stabilizing mutation. The pathogenicity of these mutations was also evaluated and compared with the predicted unfolding. Our unfolding values were then compared to 27 RPE65 mutations in which 11-cis retinol production was measured^46^. Using the same binary system, we found 66% agreement between the values (Supplemental Table S4).

## Discussion

We created a program for complete mutation scans of protein unfolding, for use in the *in silico* evaluation of the severity of genetic perturbations at the atomic level of protein structure. The tool has the new feature to predict the effect of every possible missense mutation on protein folding. The quality of the unfolding calculations was verified by experiments on 1391 mutant variants from 16 crystal protein structures. On average, each protein from the validation dataset had 77.9 ± 9.1% correct matches between the experimental and computed unfolding. Afterward, UMS was applied to evaluate the role of patient genetic changes in several degenerative eye disorders, such as AMD, adRP, and LCA.

**Figure 6:**
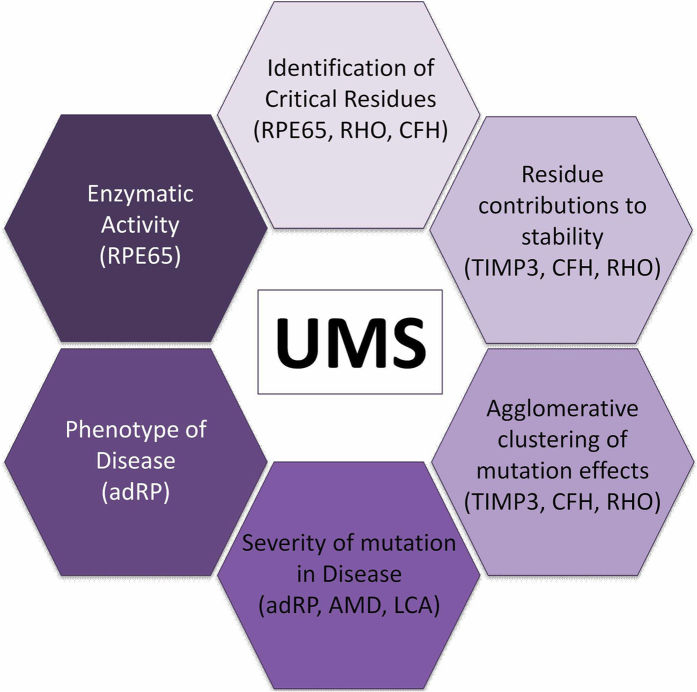
Applications of the UMS method. The combined knowledge of genetic mutations and atomic protein structures creates a diverse group of applications for the UMS method. We show six of the applications explored in our study and the corresponding disease-related proteins involved in these applications.

In this work, the UMS method was created to predict the outcomes of all possible single missense changes in a protein structure. Traditionally, most of computational methods analyze an absolute or relative free energy changes (ΔΔG) with a purpose to evaluate the effect of mutation in a single amino acid^18^,^19^,^20^,^21^,^22^,^23^. Typically, ΔΔG values have a wide range from negative values (stabilizing mutations) to higher positive values (destabilizing mutations). Here we are using a new approach based on the analysis of mutation effects from the protein-unfolding curve to evaluate the unfolded protein fraction. UMS calculates an unfolding propensity, which is derived from the protein-unfolding curve and can be used to classify the effect of genetic mutations on the folding of a protein and ultimately its functional ability. The unfolded protein fraction value is a positive number ranging from 0 to 1, which correspond to the folded and fully unfolded protein. In contrast to using the ΔΔG values, this approach provides a better indicator for the cumulative analysis of several mutations in the same protein structure to evaluate the unfolding effect. This ability leads to a number of applications in the analysis of the effect of missense mutations on protein structure (Fig. 6). Various methods could be used to compute the Gibbs free energy changes (ΔΔG) of mutant proteins^29^,^47^,^48^,^49^,^50^,^51^. We select our ΔΔG calculation method based on previous studies comparing various tools^13^. Although the Gibbs free energy change (ΔΔG) of a mutant protein can accurately describe the protein stability and therefore its ability to fold properly^52^, no program existed that could compute all possible unfolding propensities and use these propensities to identify critical residues in the protein structure.

UMS differs from previous predictors in that it predicts protein folding based on thermodynamic data. UMS can identify mutations that will cause the protein to misfold. Previously, alanine scans have been used to predict the functional roles of protein residues. Mutations to alanine are used in these scans because they do not alter the main chain of the protein, impose electrostatic or steric effects, or eliminate the side chain beyond the beta carbon^53^. Although alanine scans could be performed computationally in FoldX, UMS provides a more thorough analysis than an alanine scan by considering all possible missense mutations and their effects on protein folding. Thus, we are able not only to identify critical residues but also to see which residues, when introduced as a mutation, have the greatest and least effects on protein stability.

UMS provides a number of benefits and advances over current mutant screening techniques. Because UMS is derived from the atomic structure level and thermodynamics rather than sequence conservation, UMS can predict the effects of *de novo* missense mutations. A common challenge in current computational tools is interpreting data in terms of functional effects^24^; UMS addresses this challenge by using the unfolding propensity to determine the functional ability of the protein. If a protein is not folded properly, it may result in a complete loss of function, a partial loss of function, or even a gain of function^54^. In our method not only is the unfolding propensity scale allows us to differentiate between these effects, but it is a universal value that can be compared across proteins rather than being related to a specific protein. UMS also reduces errors in molecular modeling, a common concern with computational methods^16^ because the internal control allows constant quality checks on the data produced from the program.

A deep mutation analysis was performed experimentally^55^,^56^,^57^, but these experimental methods are limited by the protein sequence length (300 residues), the time they require, and the cost of materials. Although the time required for the calculations for a single mutation in the UMS code and the computational accuracy are similar to the values for FoldX, the major acceleration effect of our code is achieved at the stage of the map analysis. If the protein structure is available and the maps are calculated in advance, an investigator can obtain the unfolding propensities in seconds from the interactive maps. In addition, the 3 maps are designed to make this large dataset readable for investigators with different backgrounds, who may not have any preliminary experience in homology modeling and the calculations of protein stability. The maps are precalculated and saved on a server so that little time is required to obtain the data for a particular protein. A geneticist may be interested in using the data to analyze next-generation sequencing data. A clinician could use the standard heat map to quickly and easily identify the unfolding propensity for a patient with a novel mutation. A biochemist might use the foldability structure to analyze where the most severe mutations occur in the structure. The clustered heat map could enable a pharmacologist to see which mutations have stabilizing effects on the protein structure and how they might facilitate the development of new drugs.

Compared to the experimental deep mutation scan, *in silico* unfolding shows no limit regarding the size of the protein. However, there are some limitations related to the availability of homology models or experimental protein atomic structures. These limitations are crucial for the analysis of novel human genes, some of whose pathogenic mutations could not be analyzed due to an inability to create a reliable protein atomic model. There are also other limitations in using our method. Currently, UMS can only analyze single missense changes, and the effect of several mutational changes in the same protein is difficult to visualize generally. In addition, our calculations do not consider cases where single mutations can be compensated by either neighboring side chains or backbone movements. Furthermore, this approach is best for proteins from inherited diseases that are associated with full or partial protein misfolding in the ER. UMS is less useful for analyzing the mutations at surface residues that cause no conformational changes in the native protein structure but are associated with the disruption of proper protein sorting or for analyzing the mutations that occur in proteins with no observed biochemical or cellular function.

The primary function of UMS is to identify critical residues in a protein structure and isolate a specific region of the protein for targeted experimental methods. A UMS scan looks for protein unfolding propensities without restriction to the active site of the protein and shows all areas that are essential for proper folding and the retention of function. In contrast, the SNPeffect database shows only a few select mutations with precalculated free energy changes and no unfolding predictions. In this work, ~7000 rhodopsin, ~24,000 CFH, and ~21,000 RPE65 mutations were generated using UMS to identify the residues critical for proper protein folding (52 for rhodopsin, 235 for CFH, and 70 for RPE65). The effects of these residues were compared with known disease-causing missense mutations (83.3% of 90 for rhodopsin and 71.9% of 32 for CFH) or recombinant mutant variants (38 for RPE65) and indicate a role in protein unfolding.

One of the benefits of analyzing rhodopsin is the availability of extensive phenotype data linking mutations in the protein to various eye diseases. These mutations have also been classified based on the mutated protein behaviors^42^. Most class II mutations (88.5%), causing protein misfolding, were consistent with our unfolding data and had predicted unfolding propensities averaging 0.88 ± 0.24 for experimentally classified class II mutations. These values confirm the definitions Sung *et al*. assigned to class I and II mutations, in which class II mutations are misfolded proteins retained in the ER. The unfolding value was also consistent with the disease phenotype data. In RP patients who exhibited a young age of onset, we also observed severe destabilization and predicted unfolding (Table S3).

Complement factor H is encoded by the CFH gene, and its primary function is to regulate the body’s immune response as part of the complement system. The protein is composed of 20 sushi domains, and missense mutations within the domains have been linked to age-related macular degeneration^58^. As indicated in the overlaid logo plot, our analysis of the CFH domains used multiple sequence alignment to compare unfolding effects with the most conserved residues. In CFH, the most conserved residues also exhibited the most severe unfolding effects when mutated. The conservation and foldability comparison of CFH revealed that cysteine, glycine, and proline are the most conserved residues and demonstrate the highest foldability in the sushi domains. The tryptophan in the hydrophobic core is also highly conserved. The formation of disulfide bridges in the sushi domains was predicted to be extremely important for proper protein folding.

RPE65 is produced in the retinal pigment epithelium (RPE) layer that lines the back of the eye, and it is involved in the visual cycle by converting 11-trans retinal to 11-cis retinal, prompting the restart of the visual cycle^59^. A number of mutations in RPE65 have been linked to LCA, an inherited eye disease that affects the retina. Patients with LCA have visual impairment from a young age, but the disease progresses slowly. Destabilizing RPE65 mutations are expected to decrease the enzymatic activity of the protein. These data indicate that the mutations that lead to decreased activity also have destabilizing unfolding propensities consistent with a loss of isomer hydrolysis (73%) or 11-cis retinol (66%) enzymatic activity.

Through our analysis of CFH, rhodopsin, and RPE65, we were able both to draw conclusions relating to critical residues in protein structures and to predict misfolding-causing mutations in genetic disease. These results also suggest that the unfolding value may be a good indicator of disease severity. In conclusion, UMS is a tool for predicting the unfolding effects of missense mutations on protein structure, stability, and disease phenotype. The method could be useful for protein design, the rapid analysis of missense mutation severities and genotype-to-phenotype associations in clinical studies and the analysis of next-generation sequencing data and protein structure.

## Materials and Methods

### Unfolding mutation heat maps

Figure 1 is a workflow diagram of the UMS process. We developed a script using the Python, R, and Bash programing languages to read PDB files and generate a complete list of 19 possible missense mutations for each residue in the protein structure along with an identity mutation. The output of this list yields 20 x (number of residues in the protein sequence) mutations that can then be read and processed by FoldX^29^. FoldX was used to calculate the free energy changes (ΔΔG_m_) for each mutation in the specified list. The ΔΔG_m_ values were used to calculate the unfolding propensity of the mutation and sort the resulting data into a rectangular matrix.

The unfolding propensity ranges from 0–1, where values less than 0.5 are stabilizing mutations, and values greater than 0.5 are destabilizing mutations. The value 0.5 represents the folding-unfolding equilibrium with no change in stability. The unfolding propensity is derived from the same sigmoidal unfolding curve as the ΔΔG value. This value describes the fractions of the protein in the folded, unfolded and folding-unfolding equilibrium states. In this matrix, each row (X-axis) and column (Y-axis) corresponds to the position of an amino acid in a protein sequence and one of 20 mutations, respectively.

Two different maps were calculated. First, the unfolding heat map displays the wild-type residues on the y-axis according to location within the structure, and the mutant residues lie along the X-axis in alphabetical order. Each mutation within the structure is colored according to severity, where the reddest blocks correspond to an unfolding propensity of 1, the bluest to a propensity of 0, and other colors to intermediate values of unfolding propensity. Each box also contains a number representing the unfolding propensity. Second, the clustered unfolding heat map uses an agglomerative hierarchical method^60^ to group the mutations based on similarity. A dendrogram is used to display the path of the grouping. The mutations are depicted along the X-axis, while the Y-axis represents the residues from the wild-type protein sequence. The clustering was computed using both the X- and Y-axes. Both heat maps were generated using the d3heatmap package for R.

### Protein foldability

The foldability parameter was estimated as a sum over all mutations with unfolding propensities > 0.9 for any given position of the residue in a protein sequence. An unfolding propensity of 0.9–1 for the mutation could be considered the cause of protein unfolding due to the saturation of this region in the unfolding curve. For a particular residue position, the foldability could then be used to differentiate between areas that underwent multiple severe mutations, areas with a few, and areas with none. Foldability has advantages over simply finding the average in that foldability can successfully tally all severe mutations that occur at a certain location without being influenced by less severe mutations.

The foldability parameter was used to color each residue in the ribbon representation of the protein structure. These values range from 0 to 19, where a foldability value of 19 (red) indicates that every mutation at the site is severe, while 0 (blue) indicates none of the mutations at a particular site is severe. Each residue is assigned a foldability value, and the structures are displayed using UCSF Chimera, an extensible molecular modeling system^61^. Again, the red residues exhibit severe unfolding propensities, while the blue residues demonstrate a stabilizing effect. Here, residues with foldability values greater than 17.1 (19 mutations multiplied by 0.9) are considered critical residues.

### UMS and unfolding validation

The proteins for validation analysis were selected based on the available experimental data for protein crystal structures and for the chemical unfolding/refolding data of these proteins from the ProTherm database^62^. The protein crystal structures were selected from the Protein Data Bank, PDB (http://www.rcsb.org/pdb/), to create a validation set consisting of T4 lysozyme (PDB id: 2LZM), staphylococcal nuclease (1STN), protein L (1HZ6), barnase (1BNI), ribonuclease T1 isozyme (1RN1), gene V protein (1VBQ), chymotrypsin inhibitor 2 (2CI2), acyl-coenzyme A (2ABD), tyrosine-protein kinase (1FMK), acylphosphatase (1APS), alpha spectrin (1AJ3), dihydrofolate reductase (1RK4), ribosomal protein S6 (1RIS), tryptophan synthase (1WQ5), ARC repressor (1ARR), and azurin (5AZU) for analysis^63^. For the protein structures and their mutant variants, we selected tryptophan fluorescence or CD data for chemical unfolding/refolding in the presence of urea or Gdm-HCl from the ProTherm database (http://www.abren.net/protherm/). Individual papers were then analyzed to select the appropriate unfolding thermodynamic data for use in the verification, which involved changing the signs of the values reported in ProTherm or selecting the best data for when multiple values were reported based on the experiments described. In total, the experimental unfolding propensity values were derived from the free energy changes for 1391 mutant variants. These experimental unfolding values were compared with the unfolding parameters determined *in silico*. For this purpose, each protein atomic structure was computationally mutated, and the free energy changes caused by this mutation were evaluated using the FoldX program^29^. Finally, these changes were converted to the corresponding unfolding propensities.

Several criteria were used for the comparison. First, the unfolding propensities of the mutant variants were divided into 3 groups based on their effect: stabilizing (0–0.4), folding-unfolding equilibrium (0.4–0.6), and destabilizing (0.6–1.0). The mutations from the experimental and computational data were then organized into matrices, where the frequencies along the diagonals represented matches (Supplementary Figure S1). Then, the percent matching and mismatching were calculated for each of the proteins. Second, the match quality was confirmed using a Fit Score calculated for each protein separately from the validation set. This factor shows the relative discrepancies between mutant unfolding values obtained experimentally, *Uexp*, or *in silico*, *Ucalc*. Here, 0 represents a perfect fit, while 1 is the worst fit. Finally, the Fit Score was calculated by the following formula:


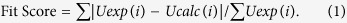


Here, the sums are calculated over all mutations for each protein in a validation group of 16 proteins.

### Internal control

The quality of the protein structures built using homology modeling and refined using molecular dynamics simulations was evaluated using a procedure called an “internal control.” This procedure verifies the overall quality of the side chain rotamers in the generated protein structure by mutating each residue from the protein sequence to itself (change to the identical residue) during a full mutation scan. In this procedure, the ΔΔG_m_ values are calculated and converted to unfolding propensities for each identity mutation. The quality of the protein atomic model was then determined by calculating the mean, standard deviation, p-value, and 95% confidence interval for the unfolding propensity values calculated over the list of identity mutations in a protein structure. It is expected that when a residue is mutated to itself, it should have an unfolding propensity of 0.5. Therefore, in our analysis, we looked for small confidence intervals centered on 0.5 with small p-values (~10^−16^). This procedure could be implemented for different frames of molecular dynamics equilibrations to select the best protein model with the lowest overall variation in unfolding propensities.

### Human proteins: rhodopsin, RPE65, and CFH domains

Atomic structures for human proteins such as rhodopsin, retinal pigment epithelium protein 65, and 4 domains of complement factor H were generated by homology modeling using the program package Yasara (http://www.yasara.org/). Briefly, protein crystal structures from the PDB were used as structural templates. For human rhodopsin, the structure of night blindness-causing G90d rhodopsin in complex with the Gact2 peptide was used (PDB file: 4BEY-A), with a sequence identity of 92.3% and similarity of 95.7%. The human RPE65 structure was modeled using the crystal structure of RPE65 at 2.14 Å resolution with 98.6% sequence identity and 99.4% similarity (PDB File: 3FSN-B). Four CFH sushi domains, 4, 5, 14, and 17, were modeled based on similarity to the known structures of other sushi domains. Domain 4 showed 100% identity and similarity to complement C3b in complex with factor H domains (PDB file 2WII-C). Domain 5 was built using the protein structure of a complex between complement control protein modules 6 and 7 of human CFH and *Neisseria meningitidis* Fhbp variant 3 wild type (PDB file: 4AYI-E), with 32.1% sequence identity and 37.5% similarity. Domain 14 was modeled using the structure of the two C-terminal domains of CFH-related protein 2 (PDB File: 3ZD1-A), with the 35.1% sequence identity and 52.6% similarity. Domain 17 was built using the same structural template but with different sequence identity (31.6%) and similarity (35.6%). The proteins were equilibrated using 1 ns molecular dynamics in water in the Yasara program package. In addition, Promals3D (http://prodata.swmed.edu/promals3d/promals3d.php) was used to align each of the sushi domains (Fig. 2a). Based on this alignment, WebLogo (http://weblogo.berkeley.edu/logo.cgi) was used to construct the logo plot for. The extended UMS library and data descriptors for 15 proteins from inherited eye disease is available from McCafferty & Sergeev (ref. 31).

## Additional Information

**How to cite this article**: McCafferty, C. L. and Sergeev, Y. V. *In silico* Mapping of Protein Unfolding Mutations for Inherited Disease. *Sci. Rep.* **6**, 37298; doi: 10.1038/srep37298 (2016).

**Publisher's note:** Springer Nature remains neutral with regard to jurisdictional claims in published maps and institutional affiliations.

## Supplementary Material

Supplementary Information

## Figures and Tables

**Table 1:** The *in silico* unfolding propensities agree with the propensities derived from the experimental free energies for 16 protein structures.

Protein	PDB	Fit Score	%Matching	%Mismatching	Mutations
T4 Lysozyme	2LZM	0.156	78.6	21.4	84
Tyrosine-Protein Kinase	1FMK	0.241	71.4	28.6	49
Barnase	1BNI	0.245	72.7	27.3	140
Staphylococcal Nuclease	1STN	0.186	74.9	25.1	521
Protein L	1HZ6	0.152	75.4	24.6	57
Ribonuclease T1 Isozyme	1RN1	0.200	68.4	31.6	38
Gene V Protein	1VQB	0.254	69.6	30.4	92
Chymotrypsin Inhibitor 2	2CI2	0.157	78.2	21.8	78
Acyl-Coenzyme A	2ABD	0.199	83.9	16.1	31
Acylphosphatase	1APS	0.168	85.7	14.3	21
Alpha Spectrin	1AJ3	0.195	66.7	33.3	63
Dihydrofolate Reductase	1RX4	0.194	80.7	19.3	57
Ribosomal Protein S6	1RIS	0.094	100	0	16
Tryptophan Synthase	1WQ5	0.347	76.3	23.7	38
ARC Repressor	1ARR	0.230	70.1	29.9	77
Azurin	5AZU	0.085	93.1	6.9	29
Total					1,391

The unfolding propensities were calculated from the experimental ΔΔG values reported in the ProTherm database^61^. The percent matching values were calculated from the match matrices in Supplementary Material Table S1. The fit score was then calculated from the raw unfolding data. The fit score ranges from 0–1, with 1 being the worst fit and 0 being a perfect fit. The number of mutations for each analyzed protein was recorded in the last row to determine the total number of mutations compared.

**Table 2:** Relationships between rhodopsin unfolding propensity, mutation class, and clinical phenotype in adRP for the selected group of pathogenic mutations, the *in silico* unfolding propensity, the mutational class^40^,^41^, and phenotype data.

Mutation	Predicted Unfolding	Class [38,39]	Avg. NB Onset [40]	Avg. VFL Onset [40]	Avg. VAL Onset [40]	Sample Size
N15S	0.78		21 ± 3.6	21.7 ± 2.9	72.5 ± 3.5	3
T58R	0.89	II	14.3 ± 7.2	37 ± 23	35 ± 8.7	4
G106R	0.99		32.2 ± 14	33 ± 13	37 ± 6.1	7
P170R	0.99		15.7 ± 1.6	17 ± 2.8	—	7
P171L	1.00	II	10 ± 0	8.3 ± 3.5	30.5 ± 10.6	4
Y178C	0.99		10.3 ± 4.5	—	—	3
G182S	1.00	II	9.8 ± 2.4	24.3 ± 18	—	4
G188R	1.00	II	9.5 ± 8.9	25.4 ± 7.1	30.3 ± 5.6	6
D190Y	1.00	II	13.1 ± 5.8	17.9 ± 6.0	34.6 ± 10.8	13
P347L	0.98		8.7 ± 4.5	10.2 ± 4.8	22 ± 10.5	24

Phenotypes were the onsets of night blindness (NB), visual field loss (VFL), and visual acuity loss (VAL). For each phenotype, the average and standard deviation were calculated using the patient data^42^ if the mutation was found in > 2 patients.
