# Supplementary material for: In silico Mapping of Protein Unfolding Mutations for Inherited Disease
Source: Sci Rep. 2016 Dec 1;6:37298. doi: 10.1038/srep37298 (PMC5131339; doi:10.1038/srep37298)
Supplement: Supplementary Information [file srep37298-s1.pdf]

In silico Mapping of Protein Unfolding Mutations for Inherited Disease

Caitlyn L. McCafferty and Yuri V. Sergeev

Supplementary Materials

Table S1: Internal control for identical change data for rhodopsin, RPE65, CFH, and TIMP3. The unfolding propensities for mutations to identical residues were used to evaluate the accuracy of the calculations. For the internal control each residue in protein sequence is mutated to itself. The expected unfolding value for this mutation is 0.5. For each of the proteins analyzed in the internal control was recorded. A student's 2 tailed t-test was used to evaluate the data. The mean, confidence interval and P-value reflect the accuracy and significance of the internal control.

Protein	Degrees of Freedom	Mean ± SD	95% Confidence Interval	P-value
Rhodopsin	694	0.49±0.02	(0.488, 0.493)	< 2.2 e -16
RPE65	1064	0.49±0.04	(0.490, 0.495)	< 2.2 e -16
CFH	1234	0.53±0.15	(0.523, 0.540)	< 2.2 e -16
TIMP3	197	0.49±0.02	(0.489, 0.495)	< 2.2 e -16

Table S2: The critical residues are presented for CFH, TIMP3, rhodopsin, and RPE65. CFH was divided into its 20 sushi domains (S1-S20) and critical residues were determined individually for each sushi domain. Residues with foldability values greater than 17.1 are considered to be critical to correct protein

folding. These values indicate that when the wild type residue is replaced with any other residue, the structure misfolds and are therefore necessary for proper protein folding.

Protein (Domain)	Critical Residues
**CFH (S1)**	G35, G45, A48, Y50, C52, G55, Y56, G69, W71, C80
**CFH (S2)**	C85, G86, P88, G89, G94, L98, G100, G101, F104, G107, C114, G122, C129, G133, W134, P139, C141
**CFH (S3)**	V144, C146, P152, G155, C178, G181, G186, M190, C192, G196, W198, P203, C205
**CFH (S4)**	C210, V215, G218, P220, Y227, F233, Y235, C237, G240, C251, G255, W256, P258, P260, C262
**CFH (S5)**	C267, G275, D276, G287, Y292, C294, G297, C309, G313, W314, P316, P318, C320
**CFH (S6)**	P324, C325, G333, G334, L335, F345, P346, G350, Y355, C357, P364, G366, C374, G378, W379, P381, P384, C385
**CFH (S7)**	C389, F391, P392, G397, G403, F406, G409, C416, G419, L422, V429, C431, G435, W436, P438, P440, C442
**CFH (S8)**	C448, G458, I460, Y467, C477, G480, C494, G498, W499, P503, C505
**CFH (S9)**	P512, F514, A517, F562, L532, Y534, H537, D538, N543, T547, S550, I551, G554, N556, D560, L561, P562, I563, C564, Y565, E566, R567
**CFH (S10)**	C569, L578, P580, Y587, G590, L593, F595, C597, G605, V609, C611, G615, L616, P618, P621, C623
**CFH (S11)**	C630, G631, P633, P634, L636, G639, G650, V655, Y657, C659, F663, I671, C673, W678, P682, C684
**CFH (S12)**	C691, G692, P695, L697, G700, P708, G712, V715, F717, C719, G727, I731, C733, G736, P742, C744
**CFH (S13)**	C753, I759, L761, N767, F771, I777, C781, G783, G786, C792, G795, E800
**CFH (S14)**	C811, P812, P815, Y829, G832, C839, C853, G856, W858, P862, C864
**CFH (S15)**	C870, P873, P874, G879, N882, G894, L897, C901, G904, F905, I907, C915, G918, W920, P923, P924, C926, G928
**CFH (S16)**	P930, C931, P934, P935, G940, V942, G952, V955, C959, G962, A971, C973, G975, P981, S983, C984
**CFH (S17)**	C989, P993, F995, A998, P1000, Y1008, G1011, Y1016, C1018, G1026, C1032, W1037, P1041, C1043
**CFH (S18)**	C1048, Y1067, P1068, G1070, Y1075, C1077, G1085, V1089, C1091, G1094, W1096, P1100, C1102
**CFH (S19)**	G1107, C1109, P1112, P1113, G1118, Y1128, Y1136, C1138, G1146, I1150, C1152, G1155, W1157, P1161, C1163
**CFH (S20)**	C1167, M1174, I1179, G1194, V1197, F1199, Y1177, G1204, Y1205, C1218, G1221, Y1225, P1226, C1228
**TIMP3**	G17, C24, C26, P31, Q32, F35, C36, V41, G55, V61, Y62, I64, Y70, R71, G72, I82, T84, C91, G92, L93, L95, Y102, L103, G106, G111, M113, G116, C118, V121, W124, G134, L135, G142, C143, C145, I147, C150, C155, C163, W165, D167, M168, G176, C184, G190, C192, W194, G197
**RHO**	G3, G6, V11, P12, G18, V20, P23, F24, P27, Q28, L31, P34, A41, M44, L47, G51, V61, L68, P71, L76, A80, A82, M86, V87, G89, G101, G106, C110, G114, L119, W126, V129, L131, R135, A153, T160, A164, A168, P170, P171, G174, W175, Y178, P180, E181, G182, L183, C187, G188, D190, Y191, Y192, F203, V204, P215, L226, M253, M257, Y268, A272, G280, P285, M288, P291, A299, P303, F313, G329
**RPE65**	G528, G484, G140, G48, G32, G40, G428, R81, G46, P47, P256, F229, P523, F196, P363, G436, G53, G286, G176, G10, G322, E21, P405, R366, G187, G438, N316, P371, N310, P211, W268, W37, W331, W402, S307, P35, P210, P181, G272, P138, P157, G496, Y431, T246, L505, L160, L370, Y204, G193, E254, L66, L403, P361, Y501, R446, Y12

Table S3: Phenotype-genotype data for 90 rhodopsin mutations linked to retinal disease. The table identifies the specific disease each mutation is linked to, the pathogenicity of the mutation, the mutational class [1, 2], the region of rhodopsin the mutation occurs in, and phenotype data available and the *in-silico* unfolding propensity. The stability classification was obtained from the unfolding propensity. The disease and pathogenicity classification data comes from ClinVar database (https://www.ncbi.nlm.nih.gov/clinvar/?term=RHO%5Bgene%5D). In the last column NB, VFL, and VAL represent night blindness, visual field loss, and visual acuity loss onsets, respectively. Here, the patient age of onset is given, Y indicates that the phenotype is present but there is not a documented age, N indicates the phenotype is not yet present in the patient.

**Mutation**	**Disease**	**Clinical Significance**	***In-silico* Unfolding**	**Stability**	**Class**	**Region**	**Phenotype (age of onset, y/o)**
T4K	RP	-	0.53	Neutral		Intradiscal	
N15S	RP4, RP	Pathogenic	0.78	Destabilizing		Intradiscal	NB : 18, 25, 20<br>VFL: 20, 25, 20<br>VAL:45, 75, 70 [3]
T17M	RP4	Pathogenic	0.11	Stabilizing	II	Intradiscal	NB:35 VFL: 40<br>VAL:35 [3]
P23A	RP4	Pathogenic	0.99	Destabilizing		Intradiscal	
P23H	RP4	Pathogenic	1	Destabilizing	II	Intradiscal	
Q28H	RP	-	1	Destabilizing		Intradiscal	NB: Y VFL: 14<br>VAL: N(52) [3]
L40R	RP	-	0.98	Destabilizing		Transmembrane	
M44T	RP	-	0.99	Destabilizing		Transmembrane	ND: 15 VFL: 20<br>VAL: 26 [3]
F45L	RP4	Pathogenic	0.61	Destabilizing		Transmembrane	
L46R	RP	-	0.4	Neutral		Transmembrane	
G51A	RP	-	0.83	Destabilizing	II	Transmembrane	
G51R	RP4	Pathogenic	1	Destabilizing	II	Transmembrane	
G51V	RP	-	1	Destabilizing	II	Transmembrane	
P53R	RP4	Pathogenic	0.76	Destabilizing		Transmembrane	
T58R	RP4	Pathogenic	0.89	Destabilizing	II	Transmembrane	ND: 10, 12, 25, 10<br>VFL: 51, 50, Y, 10<br>VAL: 40, 40, 25,<br>[3]
V87D	RP4	Pathogenic	1	Destabilizing	II	Transmembrane	
G89D	RP4	Pathogenic	1	Destabilizing	II	Transmembrane	
G90D	CSNB, Dominant 1	Pathogenic	1	Destabilizing		Transmembrane	
T94I	CSNB, Dominant 1	Pathogenic	0.38	Stabilizing		Transmembrane	
G106R	RP4	Pathogenic	0.99	Destabilizing		Intradiscal	NB:<br>9,45,41,34,32,N(8),<br>Y VFL: 12, 45, 42,<br>34, 32, N(8), Y<br>VAL: 32, 45, 42,<br>34, 32, N(8), NA<br>[3]
G106W	RP4	Pathogenic	0.99	Destabilizing	II	Intradiscal	
C110Y	RP4	Pathogenic	1	Destabilizing	II	Transmembrane	
C110F	RP	-	1	Destabilizing	II	Transmembrane	
G114D	RP4	Pathogenic	1	Destabilizing		Transmembrane	
G114V	RP	-	1	Destabilizing		Transmembrane	
L125R	RP	-	0.4	Neutral	II	Transmembrane	
S127F	RP	-	1	Destabilizing		Transmembrane	
L131P	RP	-	1	Destabilizing		Transmembrane	

R135G	RP	-	1	Destabilizing		Transmembrane	
R135L	RP4	Pathogenic	0.32	Stabilizing		Transmembrane	NB: 3, 1 VFL: 3, 15 VAL: NA, 1 [3]
R135P	RP	-	1	Destabilizing		Transmembrane	
R135W	RP4, RP, RPA	Pathogenic	1	Destabilizing		Transmembrane	NB: Y, Y, Y, 17, Y, Y VFL: Y, Y, Y, Y, Y, Y VAL: Y, Y, Y, NA, Y, N(22) [3]
V137M	RP	-	0.2	Stabilizing		Transmembrane	
C140S	RP	-	0.93	Destabilizing		Cytoplasmic	
E150K	RP4, Recessive	Pathogenic	0.86	Destabilizing		Cytoplasmic	
A164V	RP	-	1	Destabilizing	II	Transmembrane	
A164E	RP4	Pathogenic	1	Destabilizing	II	Transmembrane	NB: 37, Y VFL: 30, Y VAL: 37, NA [3]
C167R	RP	-	0.64	Destabilizing	II	Transmembrane	
C167W	RP	-	1	Destabilizing	II	Transmembrane	
C167Y	RP	-	1	Destabilizing		Transmembrane	NB: 8,7 VFL: 25, 35 VAL: 30, 40 [3]
P170R	RP	-	0.99	Destabilizing		Transmembrane	NB: 15, 15, 15, 15, 15, 19 VFL: 15, 15, Y, Y, Y, 19 VAL: N(17), 44, Y, Y, Y, Y [3]
P171E	RP	-	1	Destabilizing	II	Transmembrane	
P171S	RP4	Pathogenic	1	Destabilizing	II	Transmembrane	
P171L	RP	-	1	Destabilizing	II	Transmembrane	NB: 10, 10, 10, 10 VFL: 10, 10, 3, 10 VAL: N(38), Y, N(23), Y [3]
P171Q	RP	-	1	Destabilizing	II	Transmembrane	
G174S	RP, Recessive	Pathogenic	1	Destabilizing		Transmembrane	
Y178N	RP	-	1	Destabilizing	II	Intradiscal	
Y178C	RP4	Pathogenic	0.99	Destabilizing		Intradiscal	NB: 6, 15, 10 VFL: 12, Y, Y VAL: Y, NA, NA [3]
P180A	RP	-	0.99	Destabilizing		Intradiscal	
E181K	RP4	Pathogenic	0.99	Destabilizing	II	Intradiscal	NB: 5 VFL: 6 VAL: 16 [3]
G182S	RP4	Pathogenic	1	Destabilizing	II	Intradiscal	NB: 8, Y, 8, 13 10 VFL: 45, Y, 18, NA, 10 VAL: 50, 26, 18, NA, NA [3]
Q184P	RP	-	1	Destabilizing		Intradiscal	
S186P	RP	-	0.87	Destabilizing		Intradiscal	NB: 15, 3 VFL: 15, 3 VAL: 23, 25 [3]
S186W	RP	-	0.96	Destabilizing		Intradiscal	
C187Y	RP	-	1	Destabilizing	II	Intradiscal	
G188R	RP	Pathogenic	1	Destabilizing	II	Intradiscal	NB: 27, 5, 10, 5, 7, 3, N(3) VFL: 32, 32, 15, 25, 23, N(4), N(3) VAL: 33, 36, Y, 29, 23, Y, N(3) [3]
G188E	RP	-	1	Destabilizing	II	Intradiscal	

D190G	RP4	Pathogenic	1	Destabilizing	II	Intradiscal	
D190N	RP4	Pathogenic	0.83	Destabilizing	II	Intradiscal	
D190Y	RP4	Pathogenic	1	Destabilizing	II	Intradiscal	NB: 8, 10, 4, N(4), 18, 15, 20, 7, 18, 10, 6, 19, 15, 20 VFL: 18, 14, 10, N(4), 18, Y, 18, 14, 30, 10, 12, 25, Y, Y VAL: Y, 41, 14, Y, 42, Y, 45, 30, 40, NA, NA, 30, Y, NA [3]
T193M	RP	-	0.65	Destabilizing		Intradiscal	
M207R	RP4	Pathogenic	0.9	Destabilizing		Transmembrane	
V209M	RP	-	0.4	Neutral		Transmembrane	
H211R	RP	-	1	Destabilizing	II	Transmembrane	
H211P	RP4	Pathogenic	0.98	Destabilizing		Transmembrane	
P215T	RP	-	1	Destabilizing		Transmembrane	
M216R	RP	-	0.99	Destabilizing		Transmembrane	
M216K	RP	-	0.93	Destabilizing		Transmembrane	
F220C	RP	-	0.92	Destabilizing		Transmembrane	
C222R	RP	-	0.33	Stabilizing	II	Transmembrane	
P267L	RP	-	0.88	Destabilizing	II	Transmembrane	
P267R	RP4	Pathogenic	0.62	Destabilizing	II	Transmembrane	
S270R	RP	-	0.29	Stabilizing	II	Transmembrane	
G284S	RP, Recessive	-	0.74	Destabilizing		Transmembrane	
T289P	RP	-	1	Destabilizing		Intradiscal	NB: 25 VFL: 45 VAL: 43 [3]
A292E	CSNB, Dominant 1	Pathogenic	1	Destabilizing		Transmembrane	
K296E	RP4	Pathogenic	1	Destabilizing	II	Transmembrane	
K296M	RP	-	0.17	Stabilizing		Transmembrane	
S297R	RP	-	0.39	Stabilizing		Transmembrane	
L328P	RP	-	1	Destabilizing		Cytoplasmic	
T342M	RP	-	0.18	Stabilizing		Cytoplasmic	
V345L	RP4	Pathogenic	0.21	Stabilizing		Cytoplasmic	
V345M	RP4	Pathogenic	0.22	Stabilizing		Cytoplasmic	
A346P	RP	-	0.87	Destabilizing		Cytoplasmic	
P347A	RP	-	0.86	Destabilizing		Cytoplasmic	
P347Q	RP4	Pathogenic	0.98	Destabilizing		Cytoplasmic	
P347L	RP4	Pathogenic	0.98	Destabilizing		Cytoplasmic	NB: 10, 7, 10, 10, 7, 8, 7, 10, 12, 14, 13, 15, 10, 10, 10, 20, 2, 6, 7, 4, 10, 1, 2, Y, 3 VFL: Y, 7, 10, 10, 7, 15, Y, Y, 17, 14, 15, 15, 10, NA, NA, NA, 2, N(40), 7, 4, 10, 4, 16, Y, Y VAL: Y, 24, 20, Y, Y, 24, Y, Y, 17, Y, 25, 25, 10, NA, NA, NA, Y, Y, NA, 4, 10, 35, 30, Y, 40 [3]
P347R	RP4	Pathogenic	0.98	Destabilizing		Cytoplasmic	
P347S	RP4	Pathogenic	0.99	Destabilizing		Cytoplasmic	
P347T	RP	-	0.99	Destabilizing		Cytoplasmic	

Table S4: Comparison of experimental RPE65 activity and UMS *in-silico* unfolding propensities. The red squares indicate severe unfolding or decreased protein activity. The green squares show the stabilizing and neutral unfolding values or the enhanced and slightly decreased activity. The purple squares with the letter y display the mutations in which there is agreement between the unfolding propensities and the activity, while the orange with the letter n shows the disagreement. The mutations listed are LCA disease related mutations [4], their pathogenicity is listed or the selected mutants are thought to effect chelation on production of 11-cis-retinoids [5].

Mutation	*In-silico* Unfolding	Activity (% of WT)	Pathogenicity	Agreement	Reference
G40S	1	1.65	2	y	[4]
R44Q	0.89	1.34	2	y	[4]
R91W	0.24	5.08	3	n	[4]
R91Q	0.75	1.33	2	y	[4]
T101I	0.06	1.27	3	n	[4]
Y239D	0.99	4.59	3	y	[4]
K294T	0.93	68	0	n	[4]
Y318N	0.97	5.12	3	y	[4]
N321K	0.56	127	0	y	[4]
L408P	1	5.86	3	y	[4]
A434V	0.01	110	0	y	[4]
F61L	0.82	1.02	-	y	[5]
F61Y	0.26	7.57	-	y	[5]
F61W	0.34	1.28	-	n	[5]
F103I	0.76	2.51	-	y	[5]
F103L	0.79	28.02	-	n	[5]
F103W	0.39	24.37	-	y	[5]
F103Y	0.43	20.06	-	y	[5]
T147A	0.82	12.63	-	n	[5]
T147C	0.61	20.18	-	y	[5]
T147G	0.95	30.02	-	n	[5]
T147S	0.51	84	-	y	[5]
T147W	0.64	0	-	y	[5]
T147Y	0.07	0	-	n	[5]
Y239C	0.98	0	-	y	[5]
Y239D	0.99	0	-	y	[5]
Y239F	0.79	0	-	y	[5]
Y239L	0.9	0	-	y	[5]
Y239S	1	0	-	y	[5]
Y239T	0.99	0	-	y	[5]
Y239W	0.89	0	-	y	[5]
Y275F	0.2	14.44	-	y	[5]
Y275I	0.48	0	-	n	[5]
Y275W	0.68	0	-	y	[5]
W331F	0.97	8.34	-	n	[5]
W331Q	1	0	-	y	[5]
W331L	0.96	0	-	y	[5]
W331Y	0.95	26.72	-	n	[5]

Experimental

Computational

2LZM

	0-.4	.4-.6	.6-1
0-.4	5	3	3
.4-.6	1	8	3
.6-1	5	3	52

1FMK

	0-.4	.4-.6	.6-1
0-.4	1	1	1
.4-.6	1	4	2
.6-1	0	9	30

1BNI

	0-.4	.4-.6	.6-1
0-.4	8	0	2
.4-.6	4	4	11
.6-1	7	13	89

1STN

	0-.4	.4-.6	.6-1
0-.4	11	17	29
.4-.6	6	24	34
.6-1	7	42	347

1HZ6

	0-.4	.4-.6	.6-1
0-.4	0	0	2
.4-.6	2	0	3
.6-1	2	5	43

1RN1

	0-.4	.4-.6	.6-1
0-.4	2	0	5
.4-.6	0	1	5
.6-1	1	1	23

1VBQ

	0-.4	.4-.6	.6-1
0-.4	4	1	8
.4-.6	2	1	9
.6-1	6	4	57

2CI2

	0-.4	.4-.6	.6-1
0-.4	0	0	2
.4-.6	0	3	6
.6-1	1	8	58

2ABD

	0-.4	.4-.6	.6-1
0-.4	2	0	2
.4-.6	0	0	1
.6-1	2	1	24

1WQ5

	0-.4	.4-.6	.6-1
0-.4	7	0	1
.4-.6	1	0	2
.6-1	3	2	22

1ARR

	0-.4	.4-.6	.6-1
0-.4	1	1	4
.4-.6	2	5	3
.6-1	7	6	48

1APS

	0-.4	.4-.6	.6-1
0-.4	0	0	1
.4-.6	1	0	0
.6-1	0	1	18

1AJ3

	0-.4	.4-.6	.6-1
0-.4	3	3	5
.4-.6	2	3	4
.6-1	3	5	34

1RX4

	0-.4	.4-.6	.6-1
0-.4	0	0	6
.4-.6	0	2	2
.6-1	1	2	42

5AZU

	0-.4	.4-.6	.6-1
0-.4	0	0	0
.4-.6	0	0	0
.6-1	0	2	27

1RIS

	0-.4	.4-.6	.6-1
0-.4	0	0	0
.4-.6	0	0	0
.6-1	0	0	16

Figure S1: A validation matrix was used to evaluate the accuracy of the UMS. The validation set proteins were selected from the ProTherm database [6]; their crystal structures were obtained from RCSB [7]. Experimental data for the 16 proteins in the validation set were compared to the computational unfolding values. The mutation stability was divided into 3 groups: stabilizing (0-0.4), folding-unfolding equilibrium (0.4-0.6), and destabilizing (0.6-1.0). The grey squares represent the areas in which the computational unfolding values matched the experimental data. The grey squares have the largest concentration of data validating the goodness of fit of the method to experimental data.

Figure S2: Standard and clustered heat maps for a),b) RPE65, c),d) Rhodopsin, e),f) TIMP3, and g),h) CFH (respectively)**. In each of the heat maps the mutations are along the x-axis and the wild type sequence is along the y-axis. The red squares represent the mutations with the most destabilizing effects while the blue reflects the mutations with the most stabilizing effects. The white squares signify mutations that no effect on the proteins stability. In the standard heat map, the sequence is represented in order on the y-axis. The clustered heat map uses an agglomerative hierarchal grouping method to cluster the mutations. A dendrogram is used to map the groupings on both the X and Y-axes. The standard heat map may be used for quick look up of a specific mutation or looking at specific region in the structure. The clustered heat map provides a method to identify trends in the unfolding propensities.

** The heat maps are HTML files and there is currently not a method for uploading such files. Heat maps may be provided by request. Currently a database is being created to store and access the data.

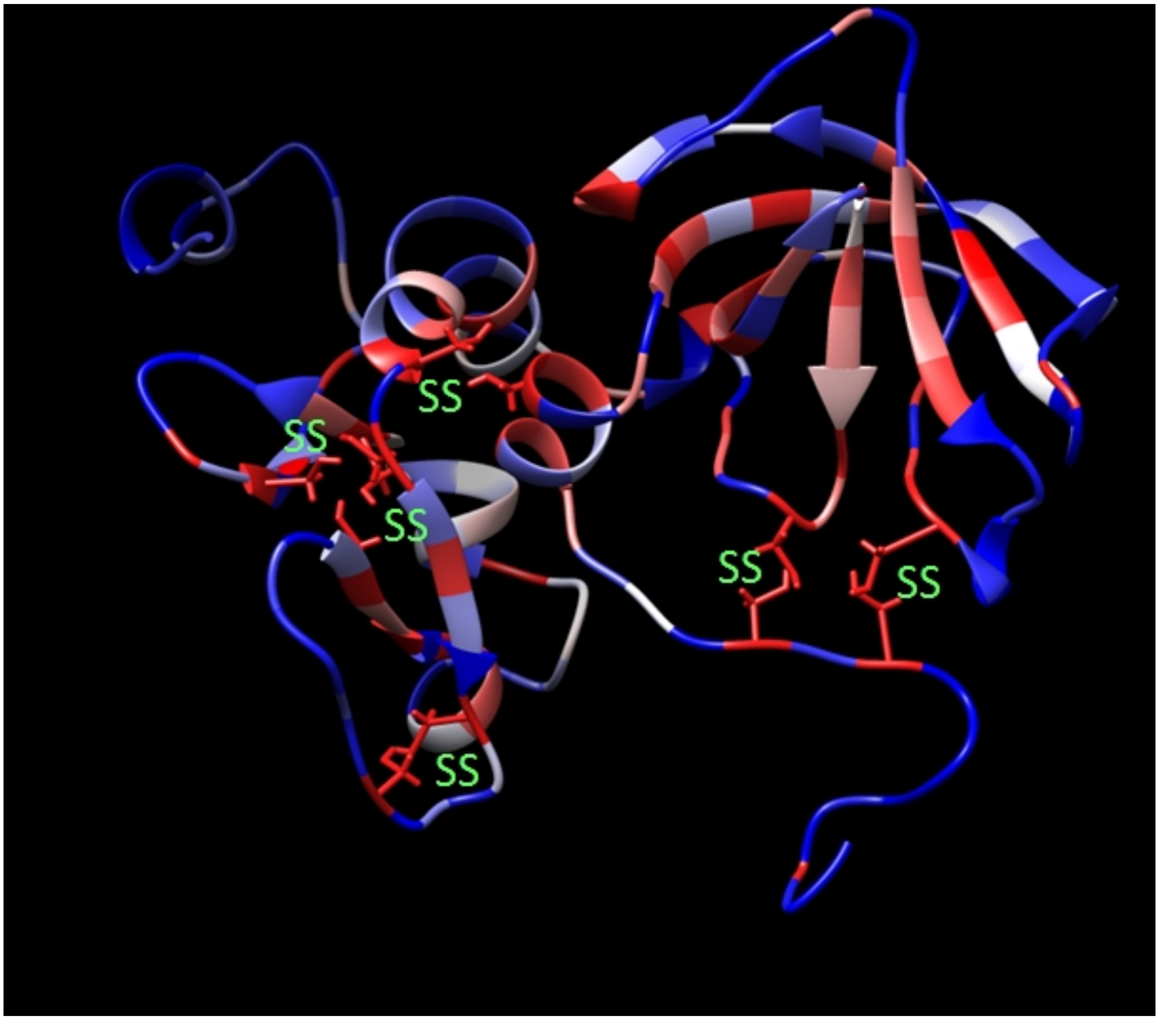

Figure S3: The foldability structure for human TIMP3. The TIMP3 structure was prepared using homology modeling. The red residues represent the wild type residues that undergo the most severe unfolding effects (high foldability) when mutated, while the blue residues show low foldability when mutated. The cysteine side chains are visible to illustrate the disulfide bonding (SS) that occurs in the structures. All cysteines in the structure are residues critical for protein folding.

References

1. Sung, C., C. Davenport, and J. Nathans, *Rhodopsin mutations responsible for autosomal dominant retinitis pigmentosa. Clustering of functional classes along the polypeptide chain.* Journal of Biological Chemistry, 1993. **268**(35): p. 26645-26649.
2. Mendes, H.F., et al., *Mechanisms of cell death in rhodopsin retinitis pigmentosa: implications for therapy.* Trends in molecular medicine, 2005. **11**(4): p. 177-185.
3. Jose, F.S., et al., *Prevalence of Rhodopsin mutations in autosomal dominant Retinitis Pigmentosa in Spain: clinical and analytical review in 200 families.* Acta ophthalmologica, 2015. **93**(1): p. e38-e44.
4. Philp, A., et al., *Predicting the pathogenicity of RPE65 mutations.* Human mutation, 2009. **30**(8): p. 1183.
5. Redmond, T.M., et al., *Mutation of key residues of RPE65 abolishes its enzymatic role as isomerohydrolase in the visual cycle.* Proceedings of the National Academy of Sciences of the United States of America, 2005. **102**(38): p. 13658-13663.
6. Bava, K.A., et al., *ProTherm, version 4.0: thermodynamic database for proteins and mutants.* Nucleic acids research, 2004. **32**(suppl 1): p. D120-D121.
7. Bernstein, F.C., et al., *The Protein Data Bank: a computer-based archival file for macromolecular structures.* Archives of biochemistry and biophysics, 1978. **185**(2): p. 584-591.
